# Incidence, prevalence, and occurrence rate of infection among adults hospitalized after traumatic brain injury: study protocol for a systematic review and meta-analysis

**DOI:** 10.1186/2046-4053-2-68

**Published:** 2013-08-24

**Authors:** Brittney NV Scott, Derek J Roberts, Helen Lee Robertson, Andreas H Kramer, Kevin B Laupland, Shalina S Ousman, Paul Kubes, David A Zygun

**Affiliations:** 1 Department of Critical Care Medicine, University of Calgary and the Foothills Medical Centre, 3134 Hospital Drive NW, Calgary, Alberta T2N 5A1, Canada; 2 Calvin, Phoebe, and Joan Snyder Institute for Chronic Diseases, University of Calgary, 3280 Hospital Drive NW, Calgary, Alberta T2N 4Z6, Canada; 3 Department of Community Health Sciences, University of Calgary, 3280 Hospital Drive NW, Calgary, Alberta T2N 4Z6, Canada; 4 Department of Surgery, University of Calgary and the Foothills Medical Centre, 1403 29th Street NW, Calgary, Alberta T2N 2T9, Canada; 5 Health Sciences Library, University of Calgary, Health Sciences Centre, 3330 Hospital Drive NW, Calgary, Alberta T2N 4N1, Canada; 6 Department of Clinical Neurosciences, University of Calgary and the Foothills Medical Centre, 1403 29th Street NW, Calgary, Alberta T2N 2T9, Canada; 7 Hotchkiss Brain Institute, University of Calgary, 3330 Hospital Drive NW, Calgary, Alberta T2N 4N1, Canada; 8 Department of Medicine, University of Calgary and the Foothills Medical Centre, 1403 29th Street NW, Calgary, Alberta T2N 2T9, Canada; 9 Intensive Care Unit, Royal Inland Hospital, 311 Columbia Street, Kamloops, British Columbia V2C 2T1, Canada; 10 Department of Cell Biology and Anatomy, University of Calgary, 3280 Hospital Drive NW, Calgary, Alberta T2N 4Z6, Canada; 11 Department of Physiology and Pharmacology, University of Calgary, 3330 Hospital Drive NW, Calgary, Alberta T2N 4N1, Canada; 12 Division of Critical Care Medicine, University of Alberta and the University of Alberta Hospital, 8440 - 112 Street, Edmonton, Alberta T6G 2B7, Canada

**Keywords:** Craniocerebral trauma, Infection, Incidence, Prevalence, Systematic review, Meta-analysis

## Abstract

**Background:**

Infection occurs commonly among patients hospitalized after traumatic brain injury (TBI) and has been associated with increased intensive care unit and hospital lengths of stay and an elevated risk of poor neurological outcome and mortality. However, as many relevant published studies to date have varied in the type and severity of TBI among included patients as well as in their design (randomized versus non-randomized), risk of bias, and setting (hospital ward versus intensive care unit), their reported estimates of infection occurrence vary considerably. Thus, the purpose of this systematic review and meta-analysis is to estimate the incidence, prevalence, and occurrence rate of infection among patients hospitalized after TBI.

**Methods/Design:**

We will search electronic bibliographic databases (MEDLINE, EMBASE, PubMed, Cumulative Index to Nursing and Allied Health Literature (CINAHL), Scopus, Web of Science, the Cochrane Central Register of Controlled Trials (CENTRAL), and the Cochrane Database of Systematic Reviews) from their first available date as well as personal files, reference lists of included articles, and conference proceedings. Two investigators will independently screen titles and abstracts and select cohort studies, cross-sectional studies, and randomized controlled trials involving adults hospitalized after TBI that reported estimates of cumulative incidence, incidence rate, prevalence, or occurrence rate of infection for inclusion in the systematic review. These investigators will also independently extract data and assess risk of bias. We will exclude studies with fewer than ten patients; experimental groups allocated to treatment with antibiotics, glucocorticoids, immunosuppressants, barbiturates, or hypothermia; and studies focused on military/combat-related TBI. Pooled estimates of cumulative incidence, incidence rate, prevalence, and occurrence rate will be calculated using random effects models. We will also calculate I^2^ and Cochran Q statistics to assess for inter-study heterogeneity and conduct stratified analyses and univariate meta-regression to determine the influence of pre-defined study-level covariates on our pooled estimates.

**Discussion:**

This study will compile the world literature regarding the epidemiology of infection among adults hospitalized after TBI. A better understanding of the role of infection will be helpful in the development of guidelines for patient management. This protocol has been registered in the PROSPERO International Prospective Register of Systematic Reviews (ID: CRD42013005146).

## Background

Infection is common among hospitalized patients and associated with substantially increased health care costs and worsened outcomes. Nosocomial infections affect approximately 2 million patients in the United States each year, increasing overall patient care costs by an estimated $4.5 to $5.7 billion [1,2]. Patients who develop an infection while in hospital are more likely to have longer hospital stays and adverse outcomes than those who do not [3-7]. Moreover, the development of a severe infection or sepsis has been associated with an increased risk of physical disability, permanent organ damage, cognitive impairment, and death [3,8,9].

Approximately 5% to 10% of patients admitted to an acute care hospital, and 9% to 37% of those admitted to an intensive care unit (ICU), will acquire one or more infections [1,10]. Patients with neurological injury, such as stroke and traumatic brain injury (TBI), appear to be particularly susceptible to infection [6,7,11-14]. Although aspiration due to a decreased level of consciousness may explain the development of pneumonia among some patients, research also suggests that catecholamines released as a result of brain injury-induced sympathetic activation may modulate cells of the immune system and induce systemic immunosuppression [15-20]. While this immune suppression may protect the brain from further inflammatory damage, it may also increase susceptibility to infection among those with acquired brain injury [16,17,19].

Among patients with stroke, a systematic review and meta-analysis reported that the pooled incidence of infection was 30%, with pneumonia and urinary tract infections being most common [7]. This study also reported that the incidence of infection for patients admitted to the ICU after stroke was significantly higher (45% versus 28%) [7]. In comparison to stroke, patients with TBI have an even higher reported incidence of infection. It is estimated that approximately 50% of patients with severe TBI develop at least one infectious complication during hospitalization [12-14,21]. Among those who develop infection, the most frequent location is the lung, with reported incidences of pneumonia ranging between 41% and 74% [12-14,21]. Moreover, sepsis has been found to affect between 10% and 41% of patients with severe TBI during hospitalization [6,13].

As patients with severe TBI (Glasgow Coma Score ≤8) have a significantly higher incidence of infection and sepsis compared to patients with mild or moderate TBI (Glasgow Coma Score >8), the risk of infection may correlate with severity of brain injury [6,11,12]. Among ICU patients, reported risk factors for infection include mechanical ventilation, presence of indwelling invasive devices, administration of immunosuppressive drugs, long-term or repeated use of antibiotics, and decreased host defenses due to poor chronic health status and/or acute disease processes [10]. It was found that patients with TBI who spent more than 7 days in an ICU had an increased risk for infection as compared to those who were not admitted or spent ≤7 days in an ICU [5].

To date, there has been no formal attempt to systematically review the published literature describing the epidemiology of infection after TBI. Therefore, it remains unknown whether the high reported estimates of infection in this population are homogenous across the literature. Moreover, many of the relevant published studies to date have varied in the type and severity of TBI among included patients as well as in their design (randomized versus non-randomized), risk of bias, and setting (hospital ward versus ICU). Thus, their reported results vary considerably. Therefore, the purpose of this systematic review and meta-analysis is to estimate the cumulative incidence, incidence rate, point prevalence, and occurrence rate of overall infection, as well as pneumonia (community-acquired, hospital-acquired, ventilator-associated), urinary tract infection, central nervous system infection, bloodstream infection, sepsis, severe sepsis, and septic shock among patients hospitalized after TBI. We also aim to synthesize and compare the incidence, prevalence, and occurrence rate of infection observed across randomized versus non-randomized studies. We will identify study and population characteristics associated with infection, define risk groups based on TBI severity (mild, moderate, severe), and identify sources of heterogeneity across these studies. A better understanding of the risk of infection among patients with TBI could assist healthcare providers in identifying patient subgroups that may benefit from preventative or early treatment efforts and may provide evidence to support priority setting for the allocation of scarce healthcare resources and research funds.

## Methods/Design

### Protocol and study overview

Methods for this systematic review and meta-analysis have been developed according to recommendations from the Preferred Reporting Items for Systematic Reviews and Meta-Analyses [22] and the Meta-Analysis of Observational Studies in Epidemiology [23] statements. We will begin by developing a comprehensive database containing all published estimates of the cumulative incidence, incidence rate, prevalence, and occurrence rate of infection among adults hospitalized after TBI. The goal of this database will be to comprehensively and critically analyze the world’s relevant literature in order to better understand the epidemiology of infection after TBI as part of a larger research program. This protocol has been registered in the PROSPERO International Prospective Register of Systematic Reviews (ID: CRD42013005146).

### Selection criteria

#### Population

The population of interest will include adult (≥16 years old) civilian patients hospitalized after TBI. TBI will be defined as “an alteration in brain function, or other evidence of brain pathology, caused by an external force” [24]. Whenever available, the comparison group will include adult (≥16 years old) hospitalized patients without TBI.

#### Outcome

The outcome will be the cumulative incidence (also known as incidence proportion), incidence rate, point prevalence (herein referred to as prevalence), and occurrence rate of overall infection, pneumonia (community-acquired, hospital-acquired, ventilator-associated), urinary tract infection, central nervous system infection, bloodstream infection, sepsis, severe sepsis, and septic shock (see below for methods detailing how these measures will be calculated). Although the definitions afforded by the Centers for Disease Control and Prevention Standardized Criteria [25] will be used whenever possible to define the above listed types of infections, we will accept alternate definitions used by authors (and these will be recorded and reported).

#### Study design

Cohort studies, cross-sectional studies, and randomized controlled trials (RCTs) will be included.

### Search strategy

The preliminary search strategy was developed by three investigators (BNVS, DJR, and DAZ) and will be subsequently refined by a librarian-scientist (HLR) by conducting iterative database queries and incorporating novel search terms once new and relevant articles are identified. We will search the following electronic bibliographic databases from their first available date without restrictions: Ovid MEDLINE, Ovid EMBASE, PubMed, Cumulative Index to Nursing and Allied Health Literature (CINAHL), Scopus, Web of Science, the Cochrane Central Register of Controlled Trials (CENTRAL), and the Cochrane Database of Systematic Reviews. The search will cover the themes TBI, infection, and incidence/prevalence. For the MEDLINE and EMBASE searches, filters for each search theme will be constructed using a combination of exploded Medical Subject Heading (MeSH)/Emtree terms and text words, each combined through use of the Boolean operator ‘OR’. The search themes will subsequently be combined using the Boolean operator ‘AND’ (see Appendix 1 in Additional File 1 for the final proposed MEDLINE search strategy and Appendix 2 in Additional File 2 for the final proposed EMBASE search strategy). Similar searches will subsequently be conducted in the remaining databases. Additional citations will be located by searching conference proceedings (The American Association of Neurological Surgeons, Neurocritical Care Society, Society of Critical Care Medicine, The American Association for the Surgery of Trauma, The Trauma Association of Canada, The Eastern Association for the Surgery of Trauma, and The Western Trauma Association), personal files, and reference lists of relevant reviews and included articles.

### Study selection

Two investigators (BNVS and DJR) will independently screen titles and abstracts to identify studies concerning infection among adult civilian patients hospitalized after TBI. Articles will be included if they meet the following criteria: 1) original research; 2) cohort, cross-sectional, or RCT study design; and 3) reported a cumulative incidence, incidence rate, prevalence, or occurrence rate of infection (or sufficient information is available to calculate an estimate). We will exclude: 1) animal studies; 2) pediatric studies; 3) case reports, case-series, case-control studies, and non-original articles; 4) studies that included fewer than ten patients; 5) treatment groups in studies investigating the effect of prophylactic antibiotics, glucocorticoids, immunosuppressants, barbiturates, or hypothermia; and 6) studies focused on military/combat-related TBI, as the results would not be generalizable to the source population of civilian patients with TBI. Studies that report composite outcomes (for example, rate of infection and mortality), without sufficient information to obtain an individual estimate of infection will be excluded. Among RCTs that meet the inclusion criteria, we will include only the placebo group (and not the treatment group) for studies examining the interventions listed above (prophylactic antibiotics, glucocorticoids, immunosuppressants, barbiturates, and hypothermia) as these treatments are known to influence the risk of infection. In contrast, we will include both the placebo and treatment groups for RCTs that meet the inclusion criteria in which the intervention is not one listed above (for example, trials examining use of calcium channel blockers or anti-epileptic drugs). A kappa (κ) statistic will be calculated to quantify the extent of inter-observer agreement on independent inclusion of articles [26]. Inclusion disagreement will be discussed and resolved by consensus or arbitration by a third investigator (DAZ).

### Data extraction

The same two investigators (BNVS and DJR) will independently extract data from eligible studies using a pre-designed and pilot tested electronic data extraction form. We will extract data on:

1) Publication details: year and language of publication and country in which the study was conducted.

2) Design: type of study (cohort, cross-sectional, RCT); study temporality (prospective, retrospective); patient enrollment (consecutive, non-consecutive); hospital setting (ICU, ward); use of Centers for Disease Control and Prevention standardized criteria [25] (yes, no, or unknown); and additional data for quality assessment (see below). Study design will be classified using the scheme developed by Oleckno [27]. Cohort studies and case series will be differentiated using the recommendations by Dekkers and colleagues [28].

3) Study participant details: patient characteristics (age, sex, median Glasgow Coma Score); number of patients enrolled in the study; number of patients with TBI; description of patients with TBI (isolated TBI or poly-trauma; blunt TBI or penetrating TBI; mild TBI (Glasgow Coma Score ≥13), moderate TBI (Glasgow Coma Score of 9 to 12), severe TBI (Glasgow Coma Score ≤8), or mixed severity of TBI); number of control patients; description of control patients (neurological or non-neurological; hospital controls, ICU controls, healthy controls, or other (if other, description of control group will be recorded)); Injury Severity Score of patients (number with major trauma (Injury Severity Score >15), number with minor trauma (Injury Severity Score ≤15); or the study’s mean/median Injury Severity Score is >15 versus ≤15); the percentage of patients who received mechanical ventilation; and the percentage of patients who received β-adrenergic receptor agonists and β-adrenergic receptor antagonists, as animal [20] and human [29] studies have suggested that treatment with β-adrenergic receptor antagonists after stroke is associated with reduced infection.

4) Data for outcome measures: All reported estimates, or sufficient information to calculate an estimate, of cumulative incidence, incidence rate, prevalence, and occurrence rate of infection (overall infection), pneumonia (community-acquired, hospital-acquired, or ventilator-associated), urinary tract infection, central nervous system infection, bloodstream infection, sepsis, severe sepsis, and septic shock among adult patients hospitalized with TBI and, if available, without TBI (control patients) in included articles will be extracted. Where available, we will also extract odds ratios or relative risks adjusted for potential confounding factors relating the differential odds or risk of infection among patients with TBI versus those without TBI.

### Quality assessment

The same two reviewers (BNVS and DJR) will also independently evaluate risk of bias among the included studies. The quality of observational studies will be assessed using the framework described by Hayden *et al.*, which evaluates study participation, study attrition, outcome measurement, and statistical analyses among prognostic studies included in systematic reviews using a simple ‘yes’, ‘partly’, ‘no’, ‘unsure’ scale [30]. As the commonly used Jadad score and Cochrane Collaboration criteria (which largely consider the adequacy of randomization, allocation concealment, withdrawals and dropouts, and other features) [31,32] are likely less relevant for the assessment of prognosis studies, the same tool will also be used to assess the quality of RCTs (with the exception of the statistical analyses category). We will also describe the following characteristics for each study included in our analysis: study temporality (prospective, retrospective); patient enrollment (consecutive, non-consecutive); the percentage of patients who received mechanical ventilation; and whether the study used Centers for Disease Control and Prevention standardized criteria for infection classification [25].

### Data synthesis

Both a narrative synthesis and, where possible, a quantitative meta-analysis of the data will be presented. Studies will be clustered according to design (randomized versus non-randomized), setting (ICU versus non-ICU), severity of TBI, and, where available, timing of determination of occurrence rate estimates. After the studies have been grouped into common clusters, their characteristics (including their specific design and study details and a description of the number and characteristics of the study participants included) will be presented in summary tables [33]. These study groupings will also be utilized to identify those studies for which occurrence rate estimates have been derived from similar enough patient populations and study designs such that a quantitative meta-analysis may be possible.

### Statistical analyses

#### Calculation of individual study estimates of cumulative incidence, incidence rate, prevalence, and occurrence rate of infection among patients hospitalized after TBI

We will begin by calculating the cumulative incidence, incidence rate, prevalence, and occurrence rate of infection among patients with (or, when available, without) TBI as reported by observational studies and RCTs. Cumulative incidence will be calculated using the following formula:

$$\begin{array}{l} \;\text{Cumulative}\;\text{Incidence}\;\\ \;\text{Number}\;\mathrm{of}\;\mathrm{new}\;\text{cases}\;\mathrm{of}\;\text{infection}\;\\ \;=\frac{\text{during}\;\text{hospitalization}\;\mathrm{or}\;\text{ICU}\;\text{stay}}{\text{Total}\;\text{population}\;\mathrm{at}\;\text{risk}} \end{array}$$

where the total population at risk will be defined as the number of adult hospitalized patients with TBI who do not have infection at the time of hospital or ICU admission in order to capture only those cases that are entirely new. Incidence rate will be determined using the expression:

$$\begin{array}{l} \;\text{Incidence}\;\text{rate }\;\\ \;\text{Number}\;\mathrm{of}\;\mathrm{new}\;\text{cases}\;\mathrm{of}\;\text{infection}\;\\ \;=\frac{\text{during}\;\text{hospitalization}\;\mathrm{or}\;\text{ICU}\;\text{stay}}{\text{Total}\;\text{person}‒\text{time}\;\mathrm{at}\;\text{risk}} \end{array}$$

where person-time will be defined as the length of hospital or ICU stay in days among adult patients with TBI. Prevalence will be defined as:

$$\begin{array}{l} \;\text{Prevalence}\\ \;\text{Number}\;\mathrm{of}\;\text{existing}\;\text{cases}\;\mathrm{of}\;\text{infection}\;\\ \;\mathrm{at}\;a\;\text{specific}\;\text{point}\;\mathrm{in}\;\text{time}\\ \;=\frac{\text{during}\;\text{hospitalization}\;\mathrm{or}\;\text{ICU}\;\text{stay}}{\text{Total}\;\text{defined}\;\text{population}\;}\\ \;\mathrm{at}\;\text{the}\;\text{same}\;\text{point}\;\mathrm{in}\;\text{time} \end{array}$$

where the defined population will be adult hospitalized patients with TBI. As it is often difficult to distinguish between truly incident versus prevalent cases, we will also estimate the occurrence rate [34] of infection, which will be calculated using the formula:

$$\begin{array}{l} \;\text{Occurrence}\;\text{rate}\;\\ \;\text{Number}\;\mathrm{of}\;\text{cases}\;\mathrm{of}\;\text{infection}\;\\ \;=\frac{\text{during}\;\text{hospitalization}\;\mathrm{or}\;\text{ICU}\;\text{stay}}{\text{Total}\;\text{patients}\;\mathrm{of}\;\text{patients}}\\ \;\mathrm{examined}/\mathrm{enrolled}\;\mathrm{in}\;\mathrm{the}\;\mathrm{study} \end{array}$$

where the patients examined/enrolled in the study will be adult hospitalized patients with TBI.

The standard error and 95% confidence interval of these proportions will then be determined using the Clopper-Pearson exact binomial method [35].

#### Calculation of pooled estimates of cumulative incidence, incidence rate, prevalence, and occurrence rate of infection among patients hospitalized after TBI

Individual incidence, prevalence, and occurrence rate estimates from observational studies and RCTs will then be pooled separately using a random effects model according to the method developed by DerSimonian and Laird [36]. Importantly, a random effects model was chosen as variability in measures of occurrence beyond chance is expected across studies. The pooled estimates obtained from these calculations will then be compared qualitatively to determine if experimental versus observational study design is associated with a different overall estimate of pooled incidence, prevalence, or occurrence rate of infection among those with TBI. We will also perform a cumulative meta-analysis, in which the estimates of infection over each year will be determined separately for RCTs and observational studies in order to determine the impact of each added study on the pooled estimates of infection over time.

#### Calculation of the pooled differential odds of infection among patients with TBI versus those without TBI

Where available, estimates of the most-adjusted prevalence or incidence odds ratio relating the odds of infection among patients with TBI versus those without TBI will be pooled using random effects models and the methods previously outlined by Ronksley and colleagues for handling variably adjusted individual study estimates [37]. Individual odds ratio estimates will only be entered into the model when the odds ratio was estimated from similar study designs and patient populations (for example, cohort studies relating the odds of infection among poly-trauma patients with TBI versus without TBI). If only relative risks are available from any of the identified studies, these measures will be converted into odds ratios using the method proposed by Deeks and Altman [38].

#### Influence and outlier analyses and examination for evidence of heterogeneity or small study effects potentially due to publication bias

We will examine heterogeneity separately in the pooled estimates obtained from randomized and observational studies using the Cochran Q (*P* value <0.05 considered significant) and I^2^ (a value greater than 50% representing at least moderate heterogeneity) statistics [31]. In the presence of statistical heterogeneity (defined as an I^2^ statistic of >25% in a pooled estimate), we will conduct subgroup analyses and univariate meta-regression (*P* value <0.10 considered significant given the low power of these tests) in order to determine the effect of study-level covariates on the estimates of cumulative incidence, incidence rate, prevalence, and occurrence rate. Study-level covariates of interest will include type of brain injury (isolated TBI versus poly-trauma); mechanism of TBI (blunt versus penetrating); severity of TBI (mild TBI (Glasgow Coma Score ≥13) versus moderate TBI (Glasgow Coma Score of 9 to 12) versus severe TBI (Glasgow Coma Score ≤8)); severity of injury (major trauma (Injury Severity Score >15) versus minor trauma (Injury Severity Score ≤15)); ICU versus non-ICU (studies in which participants are described as an ICU patient population versus studies in which participants are patients of a non-ICU hospital unit); and the percentage of participants that received mechanical ventilation (<25% versus 25% to 49% versus 50% to 74% versus ≥75%).

Influence and outlier analyses will also be conducted in order to determine whether certain studies were particularly influential on the pooled estimates of infection occurrence [39]. Studies deemed to be particularly influential on our created random effects models or to be outliers will be identified and excluded in turn from the model to determine their effect on our pooled estimates [39]. Any identified influential studies will then be reported, as will their effect on the overall pooled estimate. Small study effects potentially due to publication bias will also be assessed using the methods suggested by Begg and Egger [40,41]. Stata Statistical Software version 12.0 (StataCorp LP, College Station, TX, USA), particularly the metan and meta commands, will be used for all analyses.

## Discussion

This systematic review and meta-analysis will be performed to critically examine the world’s relevant literature on the epidemiology of post-TBI infection. Specifically, we aim to estimate the frequency of occurrence of infection among adults hospitalized after TBI by synthesizing and comparing the incidence, prevalence, and occurrence rate of infection observed across randomized and non-randomized studies. We will also identify study and population characteristics associated with infection, define risk groups based on TBI severity (mild, moderate, severe) and ICU stay, and identify sources of heterogeneity across these studies. Understanding the rates of infection among patients with TBI could help target patient subgroups that may benefit from early infection screening, prevention, and treatment efforts. Also, quantifying the burden of post-TBI infection will help guide decision-making for the allocation of scarce healthcare resources and funding. Results are expected to be publicly available near the conclusion of 2013.

## Abbreviations

CENTRAL: Cochrane Central Register of Controlled Trials; CINAHL: Cumulative Index to Nursing and Allied Health Literature; ICU: Intensive care unit; RCT: Randomized controlled trial; TBI: Traumatic brain injury.

## Competing interests

The authors declare that they have no competing interests.

## Authors’ contributions

BNVS, DJR, and DAZ formulated the research question, designed the study, developed the preliminary search strategy, and drafted the manuscript. HLR refined the search strategy by conducting iterative database queries and incorporating novel search terms. DJR designed the statistical analysis plan. AHK, KBL, SSO, and PK critically reviewed the manuscript for important intellectual content. All authors have read and approved the final version of the manuscript.

## Supplementary Material

Additional file 1: Appendix 1 Proposed MEDLINE search strategy. Click here for file

Additional file 2: Appendix 2 Proposed EMBASE search strategy. Click here for file
