# Supplementary material for: Incidence, prevalence, and occurrence rate of infection among adults hospitalized after traumatic brain injury: study protocol for a systematic review and meta-analysis
Source: Syst Rev. 2013 Aug 24;2:68. doi: 10.1186/2046-4053-2-68 (PMC3765722; doi:10.1186/2046-4053-2-68)
Supplement: Additional file 1: Appendix 1 — Proposed MEDLINE search strategy. [file 2046-4053-2-68-S1.docx]

Appendix 1

Proposed MEDLINE search strategy

1. EXP Craniocerebral Trauma/

2. EXP Cerebrovascular Trauma/

3. ((head OR crani* OR cerebr* OR brain* OR craniocerebr* OR intra-cran* OR central nervous system OR neurologic*) ADJ3 (injur* OR trauma*)).ti,ab.

4. ((subdural OR intradural OR extradural OR epidural OR intraventricular) ADJ3 (hematoma*)).ti,ab.

5. (subarachnoid h?emorrhage* OR cerebral contusion* OR brain contusion*).ti,ab.

6. 1 OR 2 OR 3 OR 4 OR 5

7. EXP Infection/

8. EXP Bacterial Infections/

9. EXP Pneumonia/

10. EXP Respiratory Tract Infections/

11. EXP Urinary Tract Infections/

12. EXP Cerebral Ventriculitis/

13. EXP Central Nervous System Infections/

14. EXP Sepsis/

15. infectio*.ti,ab.

16. pneumonia.ti,ab.

17. respiratory tract infection*.ti,ab.

18. (UTI OR urinary tract infection*).ti,ab.

19. (cerebral ventriculitis OR infectious ventriculitis OR encephalitis).ti,ab.

20. (central nervous system infection* OR CNS infection*).ti,ab.

21. ((respiratory tract OR urinary tract OR central nervous system OR CNS) ADJ3 (infect*)).ti,ab.

22. ((pulmonary OR lung* OR respiratory OR urinary) ADJ3 (infect*)).ti,ab.

23. ((infect*) ADJ3 (disease* OR complication*)).ti,ab.

24. (sepsis OR septic* OR bacter?emia).ti,ab.

25. OR/7-24

26. EXP Epidemiology/

27. EXP Epidemiologic Studies/

28. EXP Incidence/

29. EXP Prevalence/

30. (incidence OR prevalence OR occur* OR frequenc* OR proportion* OR rate* OR number* OR percent*).ti,ab.

31. 26 OR 27 OR 28 OR 29 OR 30

32. 6 AND 25 AND 31
