# Supplementary material for: Incidence, prevalence, and occurrence rate of infection among adults hospitalized after traumatic brain injury: study protocol for a systematic review and meta-analysis
Source: Syst Rev. 2013 Aug 24;2:68. doi: 10.1186/2046-4053-2-68 (PMC3765722; doi:10.1186/2046-4053-2-68)
Supplement: Additional file 2: Appendix 2 — Proposed EMBASE search strategy. [file 2046-4053-2-68-S2.docx]

Appendix 2

Proposed EMBASE search strategy

1. EXP Head Injury/

2. EXP Traumatic Brain Injury/

3. ((head OR crani* OR cerebr* OR brain* OR craniocerebr* OR intra-cran* OR central nervous system OR neurologic*) ADJ3 (injur* OR trauma*)).ti,ab.

4. ((subdural OR intradural OR extradural OR epidural OR intraventricular) ADJ3 (hematoma*)).ti,ab.

5. (subarachnoid h?emorrhage* OR cerebral contusion* OR brain contusion*).ti,ab.

6. 1 OR 2 OR 3 OR 4 OR 5

7. EXP Infection/

8. EXP Bacterial Infection/

9. EXP Virus Infection/

10. EXP Pneumonia/

11. EXP Respiratory Tract Infection/

12. EXP Lung Infection/

13. EXP Urinary Tract Infection/

14. EXP Central Nervous System Infection/

15. EXP Bloodstream Infection

16. EXP Sepsis/

17. infectio*.ti,ab.

18. pneumonia.ti,ab.

19. respiratory tract infection*.ti,ab.

20. (UTI OR urinary tract infection*).ti,ab.

21. (cerebral ventriculitis OR infectious ventriculitis OR encephalitis).ti,ab.

22. (central nervous system infection* OR CNS infection*).ti,ab.

23. ((respiratory tract OR urinary tract OR central nervous system OR CNS) ADJ3 (infect*)).ti,ab.

24. ((pulmonary OR lung* OR respiratory OR urinary) ADJ3 (infect*)).ti,ab.

25. ((infect*) ADJ3 (disease* OR complication*)).ti,ab.

26. (sepsis OR septic* OR bacter?emia).ti,ab.

27. OR/7-26

28. EXP Epidemiology/

29. EXP Epidemiological Data/

30. EXP Incidence/

31. EXP Prevalence/

32. EXP Infection Rate/

33. (incidence OR prevalence OR occur* OR frequenc* OR proportion* OR rate* OR number* OR percent*).ti,ab.

34. 28 OR 29 OR 30 OR 31 OR 32 OR 33

35. 6 AND 27 AND 34
